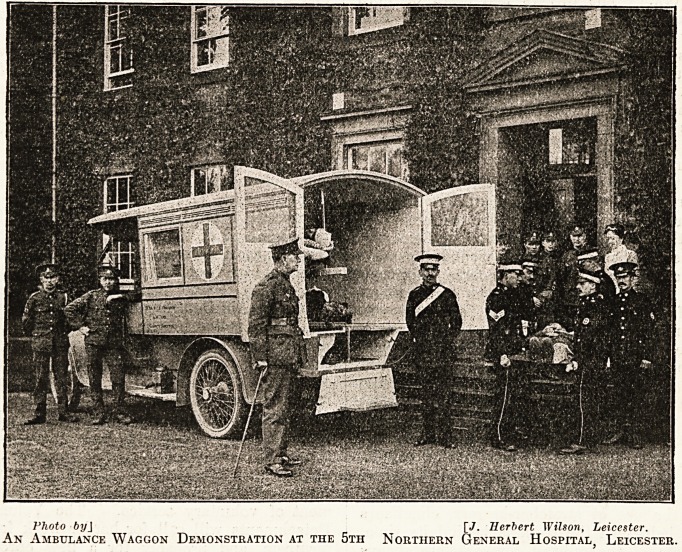# Organising a Voluntary Aid Detachment

**Published:** 1915-04-17

**Authors:** A. W. Faire

**Affiliations:** County Director of Leicester Territorial Association.


### ORGANISING A VOLUNTARY AID DETACHMENT.

### The Last Link in the Transport Service.

By A. W. FAIRE, County Director of Leicester Territorial Association.

Immediately after the publication of the scheme issued by the War Office in 1910, efforts were made to form an adequate contingent of Voluntary Aid Detachments under the Leicestershire Territorial Association. The first attempt was not altogether unsuccessful, yet the results achieved fell far short of what we desired and expected. The stimulus of immediate necessity was still lacking. The purpose of the scheme, as drafted, was to provide aid for the defenders of the country in case of invasion; and, in spite of warnings for the neglect of which we can hardly forgive ourselves, the fear of invasion was treated by the great majority of Englishmen as too remote to supply a motive for vigorous action. Those who took up the work with energy in the county of Leicester held many meetings, sought many interviews, and wrote innumerable letters; but, at the end of it all, it was with great difficulty that a provision was made of some half-dozen detachments, each consisting of fifty-six men or twenty-eight women. Very little would have been accomplished if we had not been able to enlist the help of the St. John Ambulance Association, which had been in existence in Leicester for many years, and had done excellent work in dealing with the perils of civil life; it was organised in brigades, and supplied us with volunteers already trained, and already habituated to co-operation and discipline.

As soon as the war broke out the difficulty arising from reluctance disappeared. The desire to do something to help was universal, and hundreds of men and women were eager to take advantage of an organisation which offered to them a share in definite and most useful service. New difficulties were, however, encountered. In the first place, some reorganisation was necessary to adapt a scheme, designed to operate in aid of Territorial Forces resisting invasion, to the actual conditions of warfare beyond the Channel, yet near enough to permit the repatriation of sick and wounded soldiers. In the second place, the call to the Front was urgent, and not a few of our best workers volunteered for military service, or for work on battleships or in military hospitals.

*Photo by*] [*J. Herbert Wilson, Leicester.*
An Ambulance Waggon Demonstration at the 5th Northern General Hospital, Leicester.

There was at once a great accession of zealous volunteers, some already trained and holding certificates of competence, others without experience, but eager to attend classes and acquire the necessary knowledge as rapidly as possible. It is hardly necessary to add that there has been no relapse from this early spirit of enthusiasm. The work is not easy, the call to duty comes at all hours of the day and night, and patience is often tried by long waiting in bitter weather for trains behind their time; yet it is hardly conceivable that any man—still less any woman—who has helped in the work, and has witnessed the gallant and cheerful endurance of sufferings undergone for the country's sake, should have any other feeling but a strong desire to help again; and no worker could fail to be touched to the heart by the gratitude with which his help is received and rewarded.

The Ambulance Fleet.

At our annual inspections before the war ordinary commercial vans, fitted with stretchers, were used for practice and demonstration. Eight of these vans are still in use for real work at the railway stations. It was, however, soon discovered that they were not suitable for serious cases, for which properly constructed ambulance waggons, with pneumatic tyres, are absolutely necessary. The County Director, therefore, in anticipation of an appeal to the public for funds, ordered two vans—a Daimler and a Minerva. These vans appear to be as good as anything in use for this sort of work. The owners and users of Ford cars presented a Ford ambulance van, and Earl Brownlow lent us a large omnibus, holding twenty, which has proved useful for the less serious cases. We have also a list of forty-eight closed and ninety-two open motor-cars, privately owned. These cars are at the service of the Voluntary Aid Detachments at any time, if due notice of the arrival of a train is given to the owners. A record is kept of the work done by each car.

The method of working is simple. The hospital receives from the port of disembarkation (Southampton or Dover) intimation of the despatch of a train, of the probable hour of arrival at Leicester, and of the number of sick and wounded passengers. The hospital, which, of course, relies entirely on the detachments for the transport of patients from the railway station, communicates this warning to the County Director or his secretary at the headquarters of the Territorial Association. The Director then calls up by telephone a sufficient number of stretcher-bearers, nursing sisters, and motor-cars. The nursing sisters bring with them, for the refreshment of the wounded soldiers on arrival, tea, coffee, cocoa, biscuits, chocolates, and cigarettes, all provided by private liberality. Each motor ambulance is in charge of an orderly. A waiting room at the station is set apart for the use of stretcher-bearers, chauffeurs and other helpers if the train is behind its time. The servants of the railway company are always helpful, and the station-master, Mr. Mapp, never fails to be in attendance when a train is due.

Ambulance trains are unloaded in the station yard, to which only authorised helpers are admitted. If the train is a long one it is divided on arrival into two portions, so that the walking cases and the "cot" cases can be dealt with simultaneously in different docks. The detachments have already acquired great expedition in the work of removal from the trains to the cars. A train carrying 183 patients, including forty-six "cot" cases, was unloaded in sixty-four minutes, and a train of 188, with no "cot" cases, but including some patients who could not walk by reason of frost-bitten feet, in twenty-three minutes.

Rest Stations.

In accordance with the scheme of 1910 rest stations are provided at both the railway stations (Midland and Great Central) for patients whose condition on arrival may require a pause and immediate medical attention before they are conveyed to the hospital. There are a few beds in the rest station, as well as a supply of bandages and dressings, to be used if in any case it is desirable to redress a wound without any delay. At Leicester, however, every patient hitherto has been transported to hospital without any halt at the rest station, for the distance is barely one mile, and the most careful transport need not take more than ten minutes. It has, therefore, not been thought necessary to put a nurse permanently in charge of the rest stations; but as we have reason to expect a large increase in the number of cases received, and a higher probability of patients arriving in a serious condition, we shall probably make this provision before long.

The Leicester Hospital, officially known as the 5th Northern General Hospital, contains 510 beds. In addition a considerable number of cases have from time to time been taken to the Leicester Royal Infirmary. Whenever the infirmary is used the Voluntary Aid Detachments send volunteer stretcher-bearers to that institution to act as orderlies, and we have gratifying reports of the efficiency of their work. The total number of patients transported from the stations to the hospitals since the beginning of the war is about four thousand. Our transport service also daily transfers patients from the base hospital to auxiliary hospitals.

A much larger demand on the services of Voluntary Aid Detachments, here and elsewhere, is to be anticipated in the near future. Under the new scheme of the War Office for a larger provision to be made in expectation of a great reinforcement of the troops at the Front, and of a more vigorous offensive, in April or May, the hospital accommodation at Leicester is to be increased by 2,000 beds. That is to be our local share of a total which, if we are rightly informed, will be no less than 200,000, an appalling figure, which surely implies a great increase in the requirement of voluntary service. The Voluntary Aid Detachments will call for harder work from their members, and probably for a new enrolment of helpers. In the present temper of all classes in the country we may confidently hope that, if a clear announcement of our needs is permitted, all the new help we require will be forthcoming.

Since the above was written the County Director has ordered two other motor ambulance vans—a Talbot and a Panhard. Lady Beatty, wife of Admiral Beatty, has kindly given her Mercedes chassis, and an ambulance body is being built for this.

The War Office, as already stated in The Hospital, has now taken over the North Evington Poor-Law Infirmary, which will accommodate 700 beds, also 500 more beds have been added to the 5th Northern General Hospital.

## Figures and Tables

**Figure f1:**